# Editorial: Recent trends in pharmacological treatment of musculoskeletal disorders

**DOI:** 10.3389/fphar.2022.908977

**Published:** 2022-09-02

**Authors:** Zhixian Zong, Liangliang Xu, Ning Zhang, Wing-Hoi Cheung, Gang Li, Sien Lin

**Affiliations:** ^1^ Department of Orthopaedics and Traumatology, Stem Cells and Regenerative Medicine Laboratory, Li Ka Shing Institute of Health Sciences, The Chinese University of Hong Kong, Prince of Wales Hospital, Shatin, Hong Kong SAR, China; ^2^ Orthopaedic Center, Affiliated Hospital of Guangdong Medical University, Guangdong Medical University, Zhanjiang, China; ^3^ Lingnan Medical Research Center, The First Affiliated Hospital of Guangzhou University of Chinese Medicine, Guangzhou University of Chinese Medicine, Guangzhou, China; ^4^ Department of Orthopaedic Surgery, School of Medicine, Stanford University, Stanford, CA, United States

**Keywords:** Musculoskeletal disorders, pharmacological treatment, osteoporosis, arthritis, fracture

Musculoskeletal disorders, such as osteoarthritis (OA) and osteoporosis, have already been ranked the leading cause of the global year lived with disability about 10 years ago (Collaborators, 2020). With increasing age and obesity prevalence, the morbidity of musculoskeletal disorders increased (Jin et al., 2021; Lin and Li, 2021), leading to a massive burden to the individual and society (Bitton, 2009; Collaborators, 2015; England, 2015). Pharmacological treatment becomes an effective therapeutic strategy for musculoskeletal disorders. The articles published on this Research Topic provide insight into the discoveries and applications of novel pharmacological treatments for musculoskeletal disorders.

Osteoporosis is a chronic osteolytic disease characterized by bone loss and deteriorated bone architecture, which is still challenging the research communities even though the pathology of the disease has been well elucidated. There are two main kinds of drugs for osteoporosis: anti-absorptive and anabolic drugs. Considering the cost, anti-absorptive drugs, especially Bisphosphonates (BPs), are the first-line medication in developing countries. However, the main drawback of BPs is the stiffness they bring to the bone, which may cause atypical subtrochanteric fractures (Li et al.). Antibodies targeting the pathway involved in osteoclasts activities are another option. Denosumab, an FDA-approved monoclonal antibody against the ligand of the RANK receptors, can increase the bone mineral density (BMD) of the cortical bone and decrease the porosity of the trabecular bone (Zhu et al., 2020). However, the withdrawal effect is also significant with the rapid decrease of BMD and the ensued fracture (Li et al.). Therefore, sequential application of anti-resorptive drugs is recommended to maintain the density of the bone (Li et al.). Sun et al. also reported that gene Nox4 could promote RANKL-induced bone absorption *via* activating ROS/PERK/eIF-2α/ATF4 pathway, so applying Nox4 shRNA or PERK inhibitor GSK2606414 could significantly inhibit autophagy during osteoclastogenesis. Besides, Nicorandil, which is generally used as a vasodilator drug, also showed a similar effect in inhibiting osteoclast differentiation (Xu et al.). Drugs under development are mainly focused on the adhesion process of osteoclasts to the bone and its ruffled border. Cathepsin K is the primary protease involved in the degradation of the bone matrix (Watanabe and Okazaki, 2014). Inhibiting its activities could decrease bone resorption and increase bone formation simultaneously. Data has shown that selective inhibitors of Cathepsin K, such as Odanacatib and ONO-5334, could significantly increase bone density (Li et al.). But adverse events, particularly stroke and lack of long-term study, restricted their application. Regarding anti-anabolic drugs, non-Wnt-related and Wnt-related anabolic drugs draw the most attention. Parathyroid hormone (PTH) and its related protein (PTHrP) are important local factors that regulate the bone formation. Administration of Teriparatide (a recombinant human PTH 1-34) or Abaloparatide (a synthetic analog of PTHrP) could stimulate bone formation at various sites (Li et al.). But the exact mechanism of their effect on bone density by decreasing bone resorption or increasing bone formation needs further study. Wnt signal pathways play a critical role in promoting mesenchymal stem cells committing to osteoblasts, which augment the bone regeneration (Houschyar et al., 2018). Depending on the requirement of β-catenin or not, Wnt signaling can be classified into Canonical Wnt signaling and Non-canonical Wnt signaling. Of which, canonical pathway plays the most part in bone regeneration. Sclerostin, GSK1β, and sFRP1 could be targeted to improve the therapeutic outcomes in the scenario of bone formation related to Canonical Wnt signaling. Anti-sclerostin antibodies, including Romosozumab, Blosozumab, and AbD09097, were proven to be effective in increasing BMD and decreasing the risk of fracture. Nevertheless, cardiovascular events should be closely monitored while administration, as sclerostin may also participate in the cardiovascular remodeling. Lithium and LY294002 could be used as a GSK3β inhibitor to activate the Canonical Wnt signaling pathway (Li et al.). However, both chemicals are highly toxic, and lack bone specificity, which block their way to translation. Studies on the effect of sFRP1 inhibitors on osteoporosis, including WAY-316606, miR-542-3p, miR-1-3p, were investigated, and these findings indicated that targeting sFRPs with miRNAs could be a novel way to tackle the problem (Li et al.). As a highly conserved and multifunctional signaling pathway, Wnt signaling could also interact with other signalings to regulate bone regeneration. Interestingly, Gong et al. reported that a traditional Chinese medicine formulation named *Zhuangguguanjie* could also increase mineral deposition and reduce bone resorption *via* PI3K-AKT and mTOR signaling. Tanshinol also exhibited a pro-osteogenesis effect on a glucocorticoid-induced osteoporosis rat model by reducing mitochondrial oxidative phosphorylation (Lai et al., 2021). Besides, Xie et al. reported that Kaempferol exhibited a pro-osteogenesis effect in a cell model by activating the JNK p38-MAPK signaling pathway. Periprosthetic osteolysis is a serious complication of prosthesis implantation, leading to prosthesis loosening and periprosthetic fracture. One possible cause of periprosthetic osteolysis could be the inflammation related to the wear particles released from the prosthesis. Icariin (ICA), an active ingredient of the Chinese herb *Herba Epimedii*, could significantly decrease the wear particle-induced pro-inflammatory cytokines expression and inhibit macrophage M1 polarisation (Guangtao et al.). Besides, Chrysin, a flavonoid with a wide range of anti-inflammatory functions, also showed an anti-absorption effect by inhibiting RANKL-induced osteoclastogenesis (Wu et al.). However, most of these studies only demonstrated the possibility of the compounds in ameliorating the osteolytic phenomena induced by different pathological conditions. Thus further experiments are needed to explore the underlying mechanisms.

Fracture is one of the most common causes of morbidity and mortality in the elderly, resulting in a huge and growing financial burden to our healthcare society (Wong et al., 2020). As mentioned above, bone repair depends on well-coordinated bone regeneration and absorption processes. Osteoblasts, osteoclasts, osteocytes, and precursor cells, including mesenchymal stem cells and hematopoietic stem cells, play vital roles in this scenario. Promoting the proliferation and differentiation capacity of MSCs or osteoblasts will benefit bone repair and regeneration. Yang et al. reported that a semi-synthetic derivative, Troxertin (TRX, also known as vitamin P4), improved the osteogenic differentiation capacity of human MSCs in a dose-dependent manner via stimulating the expression of β-catenin, thus accelerating the fracture healing. However, the pharmaceutical window of TRX needs further investigation as over-dose could inhibit cell viability. Besides, blood vessels are also imperative during bone regeneration, as insufficient vascularisation will impair bone regeneration. A synthetic arsenic compound named Pirfenidone (PFD) has been proven to have biphasic effects on angiogenesis depending on the dose, but the exact mechanism and dosage are not yet elucidated. Gan et al. illustrated a possible effective dose and mechanism of PFD on angiogenesis. As reported, the concentration range between 10 nM to 1 uM could significantly stimulate tube formation, and such pro-angiogenesis effects of PFD may be related to the increased expression of MMP-2/9 *via* the EGFR/p-p38 signaling pathway. As stated above, traditional Chinese medicine has shown great potential in the treatment of musculoskeletal diseases. Li et al. had summarized the recent findings of the therapeutic potential of some herbs. Among them, *Icariin*, *Ginseng*, and *Naringin* have shown multifunctions, including promoting osteogenesis, angiogenesis, chondrogenesis of the precursor cells, which are curial for tissue regeneration.

Arthritis is another common musculoskeletal disorder that leads to pain and disability. Ankylosing spondylitis (AS) is a chronic inflammatory disease characterized by the malfunction of new bone formation. Thus, manipulating the ectopic bone formation by suppressing inflammation may be feasible to slow down the progression. TNF-α and IL-17A blockers could be options for some patients, but the efficacy differed from person to person (McGonagle et al., 2021). Li et al. reported that *Danshensu* [3-(3,4-dihydroxy-phenyl) lactic acid], a bioactive ingredient extracted from *Salvia miltiorrhiza* (*Danshen*), showed an anti-osteogenic effect by suppressing the JNK and ERK signaling pathways, indicating a therapeutic potential of *Danshensu* as a novel option for AS. Besides AS, OA affects 25% of people aged >60 years worldwide. Although non-steroidal anti-inflammatory drugs (NSAIDs) or opioids can alleviate moderate to severe pain, considerable side effects still cannot be ignored, especially long-term administration is required for most patients (Leopoldino et al., 2019; Latourte et al., 2020). Disease-modifying osteoarthritis drugs (DMOADs) can be an alternative with higher efficacy and fewer side effects. Most DMOADs target cartilage degradation, synovial inflammation, and subchondral bone alteration by utilizing growth factors agonists or pro-inflammatory cytokines antagonists (Malfait and Tortorella, 2019; Latourte et al., 2020). Recently, a Wnt pathway inhibitor named Lorecivivint (SM04690) showed promising results in symptoms relieving and functional improvement in a phase II trial conducted on 700 patients for up to 2 years of follow-up Moreover, intra-articular injection of TPX-100, a novel peptide derived from matrix extra-cellar phosphoglycoprotein (MEPE), was also reported to reduce pathologic bone shape changes of the femur (Chen et al.). Interestingly, some drugs commonly used in treating other musculoskeletal disorders are also effective in treating OA. According to studies and clinical trials conducted, salmon calcitonin and Teriparatide, which are commonly targeted for metabolic bone diseases, benefit knee function. Some active ingredients in Traditional Chinese Medicine can also alleviate inflammation and OA progression. Data showed that the *Eucommia ulmoides* polysaccharides (EUP) isolated from *Eucommia ulmoides* benefited subchondral bone reconstruction and cartilage regeneration by decreasing the expression of pro-inflammatory cytokines and proportion of M1 type macrophages (Sun et al.). The antagonists of most studied pro-inflammatory related to joint inflammation had already been studied for decades, but most clinical trials failed to obtain satisfactory outcomes. To overcomes these difficulties, nucleic acid-related drugs emerged. Thanks to the development of the high-throughput genomics technologies, potential therapeutic targets of OA can now be identified by bioinformatic screening, and such technology can also facilitate us to deepen our understanding of OA. Chang et al. utilized bioinformatic screening tools to identify the key genes and miRNAs in human knee OA. They discovered that AHR, HEY1, MYC, GAP43, miR-17, miR-21, and miR-155 might contribute mainly to OA development (Chen et al.). Due to the ability to interact with miRNA and mRNA, lncRNAs, and circRNAs also play pivotal roles in the progress of musculoskeletal degenerative diseases. Among which, ncRNA/circRNA-miRNA-mRNA axis contributes greatly to disease development. Understanding their interrelationship could provide potential therapeutic strategies for musculoskeletal degenerative diseases (Zheng et al., 2021). However, till now, only a small amount of them have been discovered. Further research is needed to fill in the blanks remaining. With the rapid development of single cell “omics” technologies, researchers are now able to identify the specific cell population which mainly involved in the development of musculoskeletal diseases instead of traditional “bulk” methods. (Rai et al., 2021). By using scRNA-seq analysis, identified the trajectories and molecular mechanisms mastering cell fate of hiPSC subject to chondrocyte. devised a possible pharmacological method targeting Inf-A and Inf-D cells that could dramatically decrease inflammation in OA chondrocytes (Wu et al., 2021) (Grandi et al., 2020). Besides, Duan et al. demonstrated a rapid and reversible knockout method, proteolytic targeting chimeras (PROTACs), which could be utilized to study the target genes/proteins in a convenient and controllable way. When taken one step further, gene/non-coding RNA-based therapies provide persistent and endogenous trans-gene products which can prolong therapeutic benefits and overcome adverse effects like infection caused by multiple intra-articular drug administrations (Latourte et al., 2020). Among which, clinical trials of TNF immunoglobulin Fc fusion gene demonstrated a great improvement and well tolerance in patients (Deng et al.). Furthermore, an *ex vivo* gene therapy targeting TGF-β1 named Invossa has been approved in Korea. However, long-term follow-ups are needed, as the safety of viral vectors remains unclear. Recently, cell therapy has been intensively investigated. Some promising results were obtained in large animal models or clinical trials (Song et al., 2020; Sun A. R. et al., 2021; Zong et al., 2021). Despite encouraging outcomes acquired *in vivo* and clinical studies, severe adverse cases, including pulmonary embolism and tumors, were reported, which indicated that more randomized controlled trials are required before the final translation. Apart from trauma or aging that directly contributes to cartilage degradation, cell senescence has been found in all components of the joint, which may be closely related to the OA initiation and progression. Several anti-senescence strategies, including sirtuin-activating compounds, senolytics, and senomorphics targeted at the senescence cells and senescence-associated secretory phenotype (SASP) factors, may have beneficial effects on structural changes and symptoms (Zhang et al.). Studies have found that the synthetic STACs exhibited a higher efficacy and specificity than natural ones, and SRT1720/2104 showed promising results in reducing the expression of pro-inflammatory factors (Zhang et al.). Up till now, except Reveratrol had been driven into non-therapeutic clinical studies, most of the STACs still remain in animal studies. Another novel class of anti-senescence drugs, senolytics, also showed high effectiveness in targeting anti-apoptotic and pro-survival pathways of the senescence cells (Zhang et al.). Nogueira-Recalde et al. (2019) reported that Fenofibrate targeting on PPARα could induce apoptosis of senescent cells in cartilage and slow down the destruction of the joint in OA patients (Zhang et al.). The mitochondrion is an organelle that mainly participates in energy supplements in the cell. A recent study showed that mitochondrion also takes part in adaptive response reacted to the microenvironment changes, and dysfunctional mitochondria were believed to be closely related to senescence and cartilage degradation in OA. Thus, removing dysfunctional mitochondria or recovering their functions has been suggested as a new strategy for DMOADs development. He et al. revealed that a natural diet transformed by gut bacteria, named Urolithin A (UA), suppressed the senescence level of the chondrocytes *in vitro* and increased the quantity of extracellular matrix deposition. Despite inspiring results obtained in various studies on cell senescence, many questions remain. Senescent chondrocytes play a prior role in OA development, but cell senescence is a complex process that may be regulated by different survival pathways. Thus, more research should be made to elucidate the relationships of the signaling pathways involved to obtain a satisfying outcome.

Besides, low back pain is another common musculoskeletal disorder that severely affects the quality of life of the patients and causes worldwide productivity loss (Collaborators, 2018). Due to the innate of the disease and the weak correlation with pathology and symptoms, the diagnostic criteria and treatments are still under development, not to mention the prevention of low back pain. According to an analysis of the United Kingdom General Practice Research Database, about 2.8 billion pounds were spent on the management of the disease (Hong et al., 2013). Concerning the benefit ratio of pharmacological agents, most published guidelines advocate non-pharmacological methods including exercises and physical therapy as first-line treatments (Knezevic et al., 2021). When it comes to patients with multiple areas of pain, it leaves no choice but to utilize drugs to relieve the symptom. Similar to osteoarthritis, NSAIDs are recommended for acute or subacute low back pain according to the American College of Physicians guidelines (Qaseem et al., 2017). Administration duration, however, still requested more discussion. Because of the addictive potential, opioids were last recommended and only used when it is refractory to other drugs even such shows more efficacious than other analgesics in varieties subtypes of low back pain (Finnerup et al., 2015; Qaseem et al., 2017). Moreover, antidepressants including duloxetine and gabapentinoids are recommended to treat neuropathic pain, but evidence only supports duloxetine and gabapentinoids for chronic low back pain (Chou et al., 2017).

Taking all these together, articles on this topic demonstrate some novel tools and pharmacological methods to tackle musculoskeletal disorders, which may throw light on further studies and new drug development.
